# *Citrullus colocynthis* (L.) Schrad.: A Promising Pharmaceutical Resource for Multiple Diseases

**DOI:** 10.3390/molecules28176221

**Published:** 2023-08-24

**Authors:** Xiaotian Cheng, Minni Qin, Rongrong Chen, Yunxia Jia, Qing Zhu, Guangtong Chen, Andong Wang, Bai Ling, Weiwei Rong

**Affiliations:** 1 School of Pharmacy, Nantong University, Nantong 226001, China; 2223320015@stmail.ntu.edu.cn (X.C.);; 2 Department of Pharmacy, The Fourth Affiliated Hospital of Nantong University & The First People’s Hospital of Yancheng, Yancheng 224001, China

**Keywords:** *Citrullus colocynthis* (L.) Schrad., Cucurbitaceae, insecticide, antitumor, antidiabetic effect

## Abstract

*Citrullus colocynthis* (L.) Schrad. (Cucurbitaceae) is widely distributed in the desert areas of the world. The fruit bodies of *C. colocynthis* are recognized for their wide range of nutraceutical potential, as well as medicinal and pharmaceutical uses. The plant has been reported for various uses, such as asthma, bronchitis, cancer, colic, common cold, cough, diabetes, dysentery, and jaundice. The fruit has been extensively studied for its biological activities, which include insecticide, antitumor, and antidiabetic effects. Numerous bioactive compounds have been reported in its fruit bodies, such as essential oils, fatty acids, glycosides, alkaloids, and flavonoids. Of these, flavonoids or caffeic acid derivatives are the constituents associated with the inhibition of fungal or bacterial growth, whereas eudesmane sesquiterpenes or sesquiterpene lactones are most active against insects, mites, and nematodes. In this review, the scientific evidence for the biological activity of *C. colocynthis* against insecticide, cytotoxic, and antidiabetic effects is summarized.

## 1. Introduction

The family Cucurbitaceae, with about 123 genera and over 800 species, is found in the tropics or subtropics and is rare in temperate regions. This family of plants is generally frost-sensitive, intolerant to wet and poorly drained soils, and drought-tolerant [1,2]. The well-known members are bitter apples, cucumbers, gourds, pumpkins, and melons. Because of the increasing awareness of the health benefits of this family, their production has increased over time [3]. The genus *Citrullus* comprises annual or perennial herbaceous plants in the family Cucurbitaceae. This genus includes four species (*C. rehmii* De Winter, *C. lanatus* (Thunb.) Matsum. and Nakai, *C. ecirrhosus* Cogn., and *C. colocynthis* (L.) Schrad.) throughout tropical and South Africa, Southwest Asia, and the East Mediterranean region [4].

*C. colocynthis* (L.) Schrad., distributed in the desert areas of the world, including Sudan, Morocco, Jordan, Tunisia, and Pakistan, has nutraceutical and medicinal values [5,6,7,8,9,10,11,12]. The fruits are locally called Kattu Kattuvellari in Malayalam, colocynth/bitter in English, Pcitummatti in Tamil, Rakhal in Bengali, Anedri in Sanskrit, Indrayan in Hindi, and Hanjal in Urdu [2]. The plant is a traditional medicine used to treat asthma, jaundice, and diabetes. Recently, lots of studies have been conducted on its phytochemical compounds, pharmacology, and toxicology [13,14,15,16,17,18]. To date, there is no related review that focuses on the aspects of its insecticidal, antitumor, and antidiabetic effects. This review provides an overview of the habitat and insecticide, antitumor, and antidiabetic activities of *C. colocynthis*.

## 2. Results

### 2.1. Habitat

**Figure 1:**
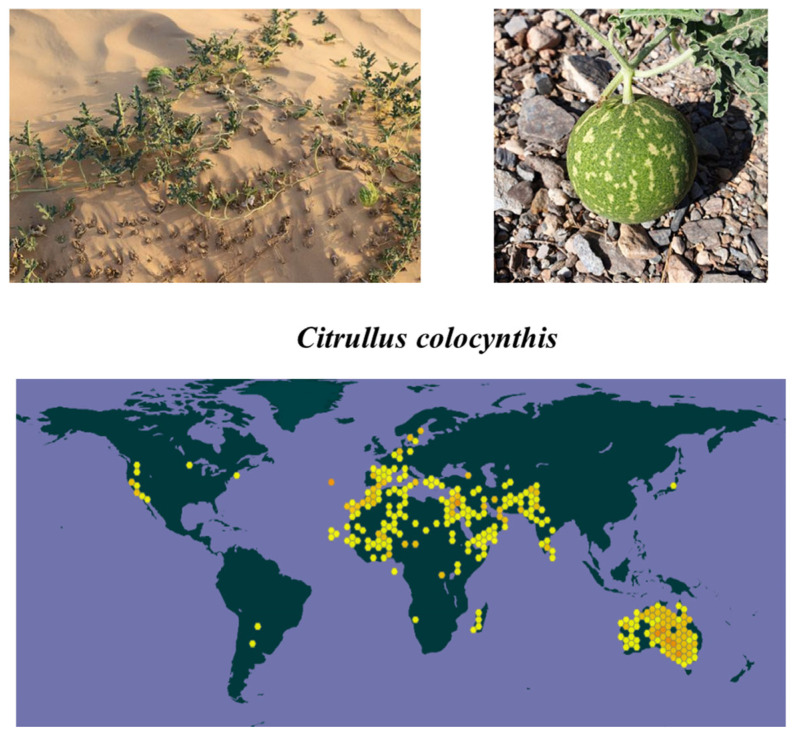
The whole plant of *C. colocynthis*, including its fruits, as well as its geographical distribution (data from the Global Biodiversity Information Facility, ”https://www.gbif.org/”).

*C. colocynthis* is a valuable plant in the family Cucurbitaceae. This plant is widely distributed in the Arabian and Sahara deserts, Sudan, and southern parts of Asia, including India, Southern Islands, and Pakistan [19,20] (Figure 1). The fruits were introduced to Spain and Cyprus by Arabs in the middle ages [21]. *C. colocynthis* is a perennial herbaceous vine that produces small flowers. The stem is rough, coarsely hairy, and angular. The leaves of this plant are alternately arranged on petioles and rough to the touch, measuring 1.5–2.0 cm in width and 5.0–10.0 cm in length. *C. colocynthis* has pale yellow and solitary flowers. The flowers are on the axils of the leaves and are yellow. They are single, pedunculated, and monecious. Each plant produces 15–30 fruits. The fruit bodies measure 7.0–10.0 cm in diameter and are green in color with undulating yellow stripes that turn yellow when dried. The fruit is globular and bitter with a smooth texture. Also, the fruit is hard and contains approximately 250 seeds/gourd. The seeds of this plant are 6 mm long, brownish, smooth, compressed, and ovoid when ripe.

### 2.2. Traditional Uses

*C. colocynthis* is widely used in many parts of the world for a number of diseases including mastitis, cancer, joint pain, jaundice, bronchitis, asthma, leprosy, constipation, and diabetes [2,5,6,7]. The clinical uses have been reported in indigenous systems of medicine in tropical and subtropical countries (Sudan, Morocco, Jordan, Tunisia, and Pakistan), which include its uses in gut disorders, such as gastroenteritis, dysentery, indigestion, and colic pain, as well as diabetes, wounds, toothache, cough, and the common cold [4,20,22,23].

In Saudi Arabia, much of the local population knows that the squeezed fruit extracts are used to elicit its purgative action, which can be achieved by treading on the fruits barefoot [5,24]. The population of Northeastern Morocco has used this plant since time immemorial to treat various cardiovascular system diseases [6]. In East Africa, seed tar is applied to the skin by nomads. But, the digestion of this fruit results in acute toxic colitis, bloody diarrhea, and changes in the colon [25]. In southern Punjab, Pakistan, the dry powder of the fruits mixed with jaggery is used in a physic drench ball [7,11,19]. In Jordan, swallowing fresh seeds, locally known as Handal, is used to treat various health issues, including its use as an abortive agent, a cathartic, a diuretic, as well as arthritis and rheumatism [8,12,26]. In southern Tunisia, *C. colocynthis* is a useful medicine for gout, arthritis, and inflammatory disorders. But, overdosing on the plant’s immature fruit is a hazard. Intoxication manifests via cerebral congestions, hypothermia, delirium, gastrointestinal irritations, and colitis [9]. In the Sariska region, the fruits are used to treat fever, general sickness, and obstructive stomachache [27]. In Eastern, Central, and Southern Iran, the fruit is recognized as an antiepileptic, abortifacient, hair growth promoter, analgesic, antidiabetic, and purgative. Adverse events (i.e., vomiting, hematochezia, diarrhea, and colic) have been associated with the use of this plant [1,28]. In Israel, the seed oils and fruits of *C. colocynthis* have been used as a laxative [2].

### 2.3. Phytochemical Analysis

The compounds of *C. colocynthis* include alkaloids, flavonoids, coumarins, steroids, and phenolic acids. The names and isolated parts of the compounds are listed in Table 1, along with the analytical methods.

**Figure 2:**
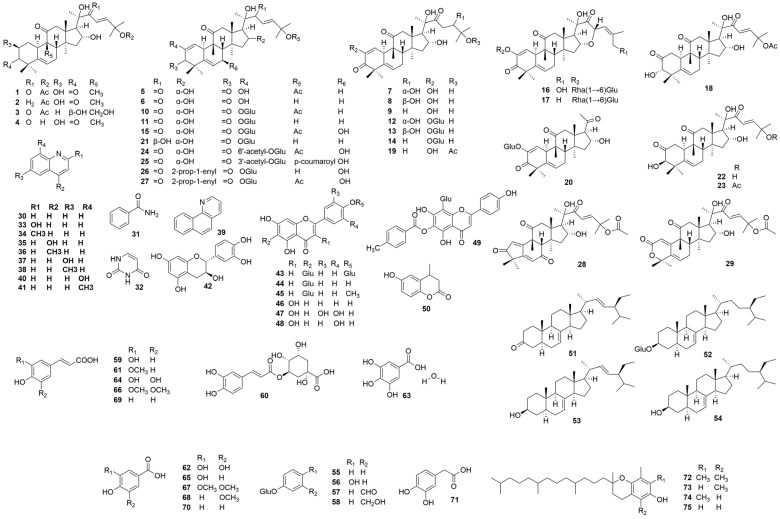
Structures of the constituents in *C. colocynthis*.

Cucurbitacins are the main constituents of this species. The serial compounds are bitter-tasting, mainly tetracyclic, highly oxygenated, derived from skeletons [19-(10→9β)-abeo-10α-lanost-5-en]. There are 12 classes of cucurbitacins according to their structure, but not all of them are present in *C. colocynthis*. Yoshikawa and Zheng et al. [4,32] systematically reported the cucurbitacins (triterpenoids and their glycosides) from this species (such as cucurbitacins A–L, and cucurbitacin E 2-O-β-D-glucopyranoside). Among these cucurbitacins, cucurbitacin E is the main component in *C. colocynthis* fruit pulp, while compound **10** is detected as the principal cucurbitacin of the fruits [29]. Besides cucurbitacins, preliminary phytochemical screening of this species shows the presence of alkaloids, flavonoids, and phenolic acids (Figure 2). Twelve alkaloids, including quinoline, nicotinamide, uracil, 2-hydroxyquinoline, 2-methylquinoline, 4-hydroxyquinoline, 4-methylquinoline, 6-hydroxyquinoline, 6-methylquinoline, 7, 8-benzoquinoline, 8-hydroxyquinoline, and 8-methylquinoline, were detected in *C. colocynthis* fruits [36]. Among these, 4-methylquinoline is an effective natural insecticide for weevils in grain storage and the management of spider mites. Apart from the principal constituents, volatile compounds, ketones, epoxy compounds, hydrocarbons [39], and fatty acids [40] are also detected in *C. colocynthis*.

### 2.4. Pharmacological Activity of C. colocynthis

#### 2.4.1. Insecticidal Activity

*C. colocynthis* is used for insecticidal activities in many countries [37,41,42,43,44]. In the results of Ahmed et al., the leaf extract of *C. colocynthis* was exceptional at controlling *Brevicoryne brassicae* L. (cabbage aphid). Cucurbitacin E [45] and spinasterol [14], isolated from this species, show strong insecticidal effects against *Aphis craccivora*. Chawech et al. [46] reported that the ethyl acetate and pure compounds (compounds **5** and **10**) showed significant larvicidal activities against *Galba truncatula* (mollusc gastropod) with the deterioration rate exceeding 89.2% and with no toxic effects against associated fauna (*Melanoides tuberculate*, *Aromia moshata*, *Hydrophilus triangularis*, and *Athous haemorhoidalis*). Elazab et al. [47] indicated that the methanol extracts of *C. colocynthis* were active against *Toxoplasma gondii* (an Apicomplexa intracellular protozoan) with an IC_50_ of 22.86 μg/mL. In Pakistan, the fruits, in combination with common or black salt, are used to treat lice infestation [48] and helminthiasis [7]. The methanol extract displays potent antimalarial activity against multidrug-resistant and chloroquine-sensitive *Plasmodium falciparum* strains, with no toxicity (IC_50_ = 6.9 and 2.01 μg/mL, respectively) [17]. The nano-extracts of *C. colocynthis* are efficient against *Trichomonas vaginalis* and safer than the drug metronidazole [49].

**Figure 3:**
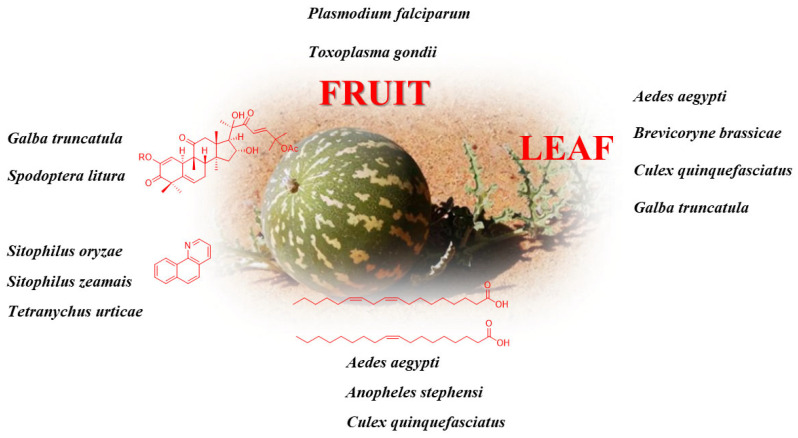
The insecticide activity of *C. colocynthis*.

7,8-Benzoquinoline isolated from fruit bodies is most effective against Tetranychus urticae. Regardless of the application method, quinoline and its structural analogs show insecticidal activities (*Sitophilus zeamais* and *S. oryzae*) [36]. Ponsankar et al. [50] reported the screening of cucurbitacin E against different larval instars and analyzed the antifeedant activity using a choice-based test. Petroleum ether shows larvicidal activity against *Aedes aegypti* L. and *Culex quinquefasciatus* Say with LC_50_ values of 74.57 and 88.24 ppm, respectively [41,51,52]. Oleic and linoleic acids are active against Culex quinquefasciatus Say (LC_50_ values of 7.66 and 27.24 ppm, respectively), Anopheles stephensi Liston (LC_50_ values of 9.79 and 11.49 ppm, respectively), and *Aedes aegypti* L. (LC_50_ value of 8.80 and 18.20 ppm, respectively) 53]. Figure 3 briefly summarizes the medicinal sites or pure compounds with insecticide activity in *C. colocynthis*, and its detailed insecticide information for different species is listed in Table 2.

#### 2.4.2. Cytotoxic Activity

**Figure 4:**
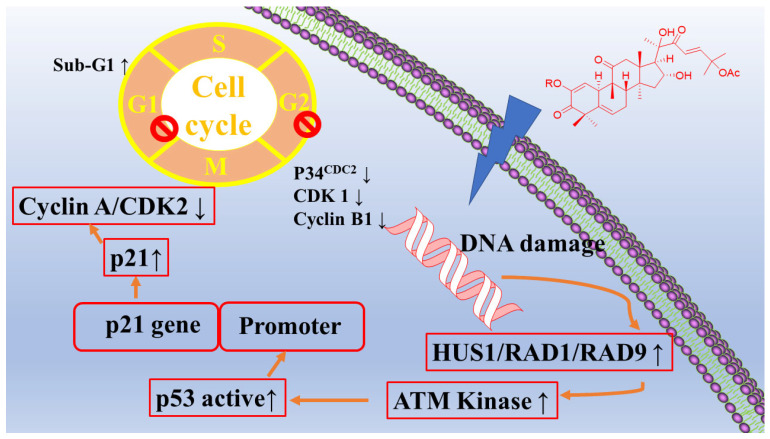
Cytotoxic activity of *C. colocynthis*.

In the professional literature, researchers have reported the antitumor activity of extracts and isolates from this species. Among them, antiproliferative effects have been observed via signaling pathways, including apoptotic pathways (inhibiting STAT3 function and increasing caspase-3) [54,55,56,57,58]. The plant extract increases the number of apoptotic cells and the proportion of sub-G1 cells [59]. The extract also promotes DNA damage in breast cancer cells via the ATM/CHK2/p53 signaling pathway (Figure 4). And, the antitumor activity of this species is achieved through cell cycle arrest [60]. The plant extract holds significant antitumor activity through the regulation of lipid metabolism (ELOVL2, ACSL5, HMGCLL1, and FASN) [61].

Ayyad et al. [23,62] reported that compounds **5** and **11** have potent inhibitory antitumor activities on HepG2, with IC_50_ values of 3.5 and 2.8 nmol/mL, respectively. The compounds also prolonged the normalization of the biochemical parameters, life span, and survival times of experimental mice. Two researchers showed cucurbitacin E and its analogs present significant cytotoxic activity against human colon cancer cell lines (HL-60, Caco-2, and HT29) [34,35]. An immunoblot analysis by Saeed et al. [63] highlighted that cucurbitacin E targets epidermal growth factor receptors (EGFRs). In a cell cycle analysis, compounds **2** and **10** resulted in the accumulation of breast cell lines (MDA-MB-231) at the G2/M phase [64,65]. In addition to cucurbitacin, linoleic acid, when compared to other oils, exhibits significant antitumor effects against colorectal cancer cells with IC_50_ values between 4 and 7 mg/mL [66]. Details of the cytotoxic activity of extracts or pure compounds from *C. colocynthis* are summarized in Table 3.

#### 2.4.3. Antidiabetic Activity

Diabetic diseases have side effects (peripheral vascular disease, stroke, nephropathy, neuropathy, and retinopathy) [6,16,24,68,69,70]. The fruit extracts possess insulin-enhancing activity [71]. *C. colocynthis* could directly reduce the formation of glycated hemoglobin (HbA1c) [13]. Benariba et al. [72,73] reported that a concentration–response correlation was observed with fruit extracts in the modulation of the insulin secretory response to D-glucose. The fruit extracts could lead to an increase in epididymal fat weight and a lesser decrease in body weight [74]. The ethanolic extract of the seeds has antioxidant and DPPH decolorization potential. It also exhibited a time-dependent decrease in blood glucose levels [75]. *C. colocynthis* seeds display a direct effect on endocrine pancreatic B cells [76].

**Figure 5:**
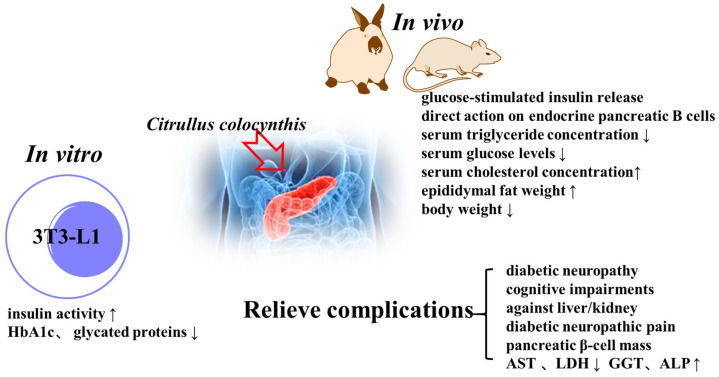
Antidiabetic activity of *C. colocynthis*.

Diabetes mellitus causes serious complications affecting multiple organs, and the literature reports the positive effects of *C. coliformis* on diabetes complications. Aqueous extracts of *C. colocynthis* ameliorate the toxic effects of streptozotocin. Oral administration of the plant extract reduced the plasma levels of aspartate aminotransferase (AST) and lactic dehydrogenase (LDH) significantly [77]. The fruit had a positive effect on the treatment of diabetic neuropathy, decreasing the number of demyelinated and degenerated nerve fibers [78]. The literature also showed the protective effects against cognitive impairments [79], pancreatic β-cell mass [80], liver/kidney [81], and diabetic neuropathic pain [82]. The antidiabetic activity of *C. coliformis* is summarized in Figure 5 and detailed in Table 4.

### 2.5. Clinical Study

*C. colocynthis* could have systemic therapeutic effects on type II diabetic patients (40 patients aged 45–65) through dermal absorption. Experiments showed that the extract reduced insulin secretion and blood glucose (BG) levels. It also decreased serum urea levels, but there was no significant change in micro-albuminuria, hepatic enzymes, lipid profiles, creatinine levels, and other related indices [83]. Barghamdi et al. showed that consumption of *C. colocynthis* extracts in the intervention group significantly reduced mean glycosylated hemoglobin and fasting blood glucose levels and did not show any side effects (≤125 mg/day). These results indicate that the aqueous extracts had hypoglycemic effects on patients with diabetes, which was associated with their saponins and glycosidic components [84]. In the research of Huseini et al. [85], a clinical experiment was conducted on 50 type II diabetic patients for 2 months. In the research of Li’s group, thirty-two type II diabetes patients (ages from 30 to 60) were arranged for this research and distributed into four groups. Capsules of different *C. colocynthis* extracts were given to patients twice a day for 30 days (1 g per day dosage) and investigated for cholesterol, triglyceride, and glucose levels. *C. colocynthis* reduced HDL, TGL, cholesterol, and glucose levels by 5, 6, 6, and 35 percent, respectively. From a clinical experiment, it was concluded that powdered *C. colocynthis* possessed good antidiabetic features [86].

### 2.6. Toxicity

The fruit of *C. colocynthis* has been used as a traditional medicine, mostly for mastitis, cancer, joint pain, jaundice, bronchitis, asthma, leprosy, constipation, and diabetes. The ingestion of this fruit, however, may have many undesired effects. The biblical story of non-fatal accidental poisoning (described in *The Book of 2 Kings*) is a related report of the medical toxicology of *C. colocynthis* in the *Old Testament* [87]. Feeding a mixture of Nerium oleander and *C. colocynthis* caused more marked effects and the death of rats [21]. In 1985, a 37-year-old Saudi man was admitted to a local hospital (Riyadh Armed Forces Hospital) with one episode of vomiting, colicky abdominal pain, and severe bloody diarrhea after he had drunk the fruit extracts of *C. colocynthis* for self-medication for indigestion. He had fresh bleeding from his rectum, and an examination revealed extreme tenderness and slight tenderness in the lower abdomen [5]. These manifestations may be accompanied by transudate in serous cavities, epicardial fat, gelatinization of renal tissue, and entero-hepato-nephrotoxicity [88].

## 3. Materials and Methods

This literature review used published scientific materials collected from the Web of Science^®^ and PubMed^®^ databases without restriction regarding the year of publication and includes literature published through July 2023. The search term used was “*Citrullus colocynthis* (L.) Schrad.”. The chemical names agree with the original references.

## 4. Conclusions and Future Perspectives

*C. colocynthis* is a valuable cucurbit plant and is widely distributed in desert regions of the world. Despite its high dietary value, *C. colocynthis* is not widely known. In our review, we systematically reviewed the research on this traditional medicine and summarized the related data on the phytochemical structure. Phytochemical studies of the species have resulted in 75 components. Of these, cucurbitacins have previously been reported as the main constituent of this species.

Crude extracts or constituents have previously been reported to have diverse pharmacological activities, with a focus on insecticide, cytotoxic, and antidiabetic effects. These surveys provide evidence of the correlations between modern pharmacological functions and ethnomedical applications in traditional Chinese medicine (TCM). Nonetheless, the extraction or composition mechanisms are not well established and merit further investigation. Besides these activities, *C. colocynthis* had other pharmacological properties, such as immune-stimulatory [89], anti-allergic [32], hypolipidemic [90,91,92], anti-microbial [32,93], reproductive [94,95], gastrointestinal tract [28,29,96], anti-inflammatory [97,98], antibacterial [31,93], and antioxidative [37,55,99] activities. Toxicology experiments and representative animal models should be used to assess their potential therapeutic effects, as mentioned in TCM.

As mentioned above, *C. colocynthis* contains a variety of chemical components, both non-volatile and volatile. Systematic purification and identification of chemical compositions in *C. colocynthis* are important. LC-MS and GC-MS is currently the most commonly used characterization technique for quickly and systematically identifying possible non-volatile and volatile components in plants [100,101,102]. However, for co-eluting compounds with similar spectra, an equivocal identification may be obtained. In recent years, some new technologies, such as gas chromatography ion mobility spectrometry (GC-IMS), have been able to provide the retention time of analytes in GC columns and the separation time of ionized compounds in IMS drift tubes, as well as the amount of each ionized compound reaching the detector in the IMS drift tube, which can significantly improve the identification of co-eluting compounds with similar spectra [103]. Therefore, the combination of LC/GC and IMS may be a good solution for the systematic identification of plants with complex chemical compositions, such as *C. colocynthis*.

## Figures and Tables

**Table 1:** A comprehensive list of the chemical compounds from *C. colocynthis*.

No.	Compound	Part of Plant	References
**Cucurbitacin and its glucosides**			
**1**	Cucurbitacin A	Fruit	[29]
**2**	Cucurbitacin B	Fruit	[29]
**3**	Cucurbitacin C	Fruit	[29]
**4**	Cucurbitacin D	Fruit	[29]
**5**	Cucurbitacin E	Fruit	[30]
**6**	Cucurbitacin I	Fruit	[31]
**7**	Cucurbitacin J	Fruit	[31]
**8**	Cucurbitacin K	Fruit	[31]
**9**	Cucurbitacin L	Fruit	[31]
**10**	Cucurbitacin E 2-O-β-D-glucopyranoside	Fruit	[32]
**11**	Cucurbitacin I 2-O-β-D-glucoside	Fruit	[32]
**12**	Cucurbitacin J 2-O-β-D-glucoside	Fruit	[32]
**13**	Cucurbitacin K 2-O-β-D-glucoside	Fruit	[32]
**14**	Cucurbitacin L 2-O-β-D-glucoside	Fruit	[32]
**15**	Colocynthosides A	Fruit	[32]
**16**	Colocynthosides B	Fruit	[32]
**17**	Deoxocucurbitoside B	Fruit	[33]
**18**	Iso-cucurbitacin B	Fruit	[33]
**19**	Dihydrocucurbitacin E	Fruit	[33]
**20**	Hexanocucurbitacin I 2-O-β-D-glucopyranoside	Fruit	[32]
**21**	Khekadaengoside E	Fruit	[32]
**22**	Dihydro-epi-iso-cucurbitacin D	Fruit	[4]
**23**	Dihydroisocucurbitacin B-25-acaetate	Fruit	[4]
**24**	6′-acetyl-2-O-β-D-glucocucurbitacin E	Leaves	[34]
**25**	25-p-coumaroyl-3′-acetyl-2-O-β-D-glucocucurbitacin I	Leaves	[34]
**26**	16-(2-prop-1-enyl)-2-O-β-D-glucopyranosyl cucurbitacin I	Fruit	[4]
**27**	16-(2-prop-1-enyl)-25-O-acetyl-2-O-β-D-glucopyranosyl cucurbitacin I	Fruit	[4]
**28**	Norcolocynthenins A	Fruit	[35]
**29**	Norcolocynthenins B	Fruit	[35]
**Alkaloids**			
**30**	Quinoline	Fruit	[2]
**31**	Nicotinamide	Fruit	[2,4]
**32**	Uracil	Fruit	[2,4]
**33**	2-hydroxyquinoline	Fruit	[2,36]
**34**	2-methylquinoline	Fruit	[2,36]
**35**	4-hydroxyquinoline	Fruit	[2,36]
**36**	4-methylquinoline	Fruit	[2,36]
**37**	6-hydroxyquinoline	Fruit	[2,36]
**38**	6-methylquinoline	Fruit	[2,36]
**39**	7,8-benzoquinoline	Fruit	[2,36]
**40**	8-hydroxyquinoline	Fruit	[2,36]
**41**	8-methylquinoline	Fruit	[2,36]
**Flavonoids**			
**42**	Catechin	Fruit	[3]
**43**	Isosaponarin	Seeds	[30]
**44**	Isovitexin	Seeds	[30]
**45**	Isoorientin 3-O-methyl ether	Seeds	[30]
**46**	Kaempferol	Fruit	[1]
**47**	Myricetin	Fruit	[1]
**48**	Quercetin	Fruit	[1]
**49**	6-*C*-p-methylbenzoylvitexin	Fruit	[1]
**Coumarin**			
**50**	6-hydroxy-4-methylcoumarin	Fruit	[22]
**Steroid and its saponins**			
**51**	α-spinasterone	Fruit	[4]
**52**	α-spinasterol-3-O-β-D-glucopyranoside	Fruit	[4]
**53**	β-sitosterol	Fruit	[4]
**54**	22,23-dihydrospinasterol	Fruit	[37]
**Aromatic rings**			
**55**	Benzyl β-D-glucopyranoside	Fruit	[32]
**56**	4-hydroxybenzyl β-D-glucopyranoside	Fruit	[32]
**57**	4-(β-D-glucopyranosyloxy)-benzaldehyde	Fruit	[32]
**58**	4-(β-D-glucopyranosyloxy)-benzal alcohol	Fruit	[32]
**Phenolic acids**			
**59**	Caffeic acid	Leaves	[22]
**60**	Chlorogenic acid	Fruit	[4]
**61**	Ferulic acid	Leaves	[22]
**62**	Gallic acid	Leaves	[22]
**63**	Gallic acid monohydrate	Roots	[22]
**64**	Hydroxycaffeic acid	Seeds	[22]
**65**	Protocatechuic acid	Fruit	[22]
**66**	Sinapic acid	Fruit	[22]
**67**	Syringic acid	Seeds	[22]
**68**	Vanillic acid	Fruit	[22]
**69**	P-coumaric acid	Leaves	[4]
**70**	P-hydroxy benzoic acid	Leaves	[22]
**71**	3,4-dihydroxyphenylacetic acid	Leaves	[22]
**Tocopherols**			
**72**	α-tocopherol	Seeds	[38]
**73**	β-tocopherol	Seeds	[38]
**74**	γ-tocopherol	Seeds	[38]
**75**	δ-tocopherol	Seeds	[38]

**Table 2:** Insecticidal activity of extracts or pure compounds from *C. colocynthis*.

No.	Active Ingredients	Scientific Species	Numerical Value	References
**1**	Fruit extracts	Helminthiasis	-	[7,48]
**2**	Fruit extracts	*Plasmodium falciparum*	IC_50_ (2.01 µg/mL)	[17]
**3**	Fruit extracts	*Toxoplasma gondii*	LC_50_ (22.86 µg/mL)	[47]
	Fruit extracts	*Haemonchus contortus*	LC_50_ (6.32 µg/mL)	[42]
**4**	Leaf extracts	*Aedes aegypti* L.	LC_50_ (74.57 ppm)	[51]
		*Culex quinquefasciatus Say*	LC_50_ (88.24 ppm)	
**5**	Leaf extracts	*Brevicoryne brassicae* L.	LC_50_(0.22 mg/mL)	[45]
**6**	Leaf extracts	*Culex quinquefasciatus*	LC_50_ (71.72 ppm)	[52]
**7**	Leaf extracts	*Galba truncatula*	LC_50_(12.6 µg/mL)	[46]
	Nano-extract	*Trichomonas vaginalis*	-	[49]
**8**	Cucurbitacin E	*Galba truncatula*	LC_50_(9.55 µg/mL)	[46]
**9**	Cucurbitacin E	*Spodoptera litura*	LC_50_ (11.58 ppm)	[50]
**10**	Cucurbitacin E 2-O-β-D-glucopyranoside	*Galba truncatula*	LC_50_(10.61 µg/mL)	[46]
**11**	Linoleic acid	*Aedes aegypti* L.	LC_50_ (18.20 ppm)	[53]
		*Anopheles stephensi Liston*	LC_50_ (11.49 ppm)	
		*Culex quinquefasciatus Say*	LC_50_ (27.24 ppm)	
**12**	Oleic acid	*Aedes aegypti* L.	LC_50_ (8.80 ppm)	[53]
		*Anopheles stephensi Liston*	LC_50_ (9.79 ppm)	
		*Culex quinquefasciatus Say*	LC_50_ (7.66 ppm)	
**13**	Spinasterol	*Brevicoryne Brassicae* L.	LC_50_(37.50 µg/mL)	[14]
	22,23-dihydrospinasterol	*Myzus persicae*	-	[37]
**14**	7,8-benzoquinoline	*Tetranychus urticae*	LC_50_ (11.8 µg/mL)	[36]
		*Sitophilus oryzae*	LC_50_ (2.9 µg/mL)	
		*Sitophilus zeamais*	LC_50_ (2.7 µg/mL)	

**Table 3:** Cytotoxic activity of extracts or pure compounds from *C. colocynthis*.

No.	Active Ingredients	In Vitro/In Vivo	Result	References
**1**	Fruit extracts	In vitro	Significant cytotoxic activity in HepG-2 and MCF-7 (LC50, 12/5430 μg/mL; LC50, 17/230 μg/mL)	[54]
**2**	Fruit extracts	In vivo	Protective drug against hepatotoxicity and nephrotoxicity together with cisplatin treatment in rats	[67]
**3**	Fruit extracts	In vivo	Negative effects on intensity, nuclear area, actin, and mitochondria, and positive effects on NF-κn	[59]
**4**	Fruit extracts	In vivo	Expression regulation of cyclin-CDK inhibitors in human breast cancer cells	[60]
**5**	Leaf extracts	In vivo	Induced apoptosis in breast cancer cells via fatty acid synthesis pathways	[61]
**6**	Seed oil fatty acid	In vitro	Significant cytotoxic activity in Caco-2 and HCT-116 (IC50, 7.1 mg/mL; IC50, 4.3 mg/mL)	[66]
**7**	Plant tinctures	In vitro	Strongest inhibition amounting to 11.8 ± 2.7% and 13.5 ± 0.5% for cell number and thymidine incorporation	[55]
**8**	Cucurbitacin B	In vitro	Activated Wnt/β-catenin signaling pathway in non-small-cell lung cancer cells	[54]
**9**	Cucurbitacin E	In vitro	Activated apoptotic pathways (inhibiting STAT3 function and increasing caspase-3) in breast cancer cells	[54,65]
**10**	Cucurbitacin E	In vitro	Targeted EGFR and silenced its downstream signaling cascades	[63]
**11**	Cucurbitacin B glucoside	In vitro	Resulted in accumulation of cells at the G2/M phase	[64]
**12**	Cucurbitacin E glucoside	In vitro	Cytotoxic activity in HepG2 hepatoma cells, with IC50 value of 3.5 µM	[23,62]
**13**	Cucurbitacin I glucoside	In vitro	Cytotoxic activity in HepG2 hepatoma cells, with IC50 values of 2.8 µM	[23,62]
**14**	25-*p*-coumaroyl-3′-acetyl-2-*O*-*β*-D-glucocucurbitacin I	In vitro	Cytotoxic activity for two human colon cancer cell lines (Caco-2 (−19%) and HT29 (−32%) at 1 µg/mL) and no cytotoxic activity for normal rat intestine epithelial cell line (IEC6)	[34]
**15**	Norcolocynthenins A	In vitro	Significant cytotoxic activity in HL-60 and PC-3 (IC50 8.32 µM and 31.26 µM)	[35]
**16**	Norcolocynthenins B	In vitro	Significant cytotoxic activity in HL-60 and PC-3 (IC50, 6.49 µM; IC50, 13.42 µM, respectively)	[35]

**Table 4:** Antidiabetic activity of extracts or pure compounds from *C. colocynthis*.

No.	Active Ingredients	Model	Result	References
**1**	Fruit extracts	3T3-L1 adipocytes	Insulin-enhancing activity	[71]
**2**	Fruit extracts	Hemoglobin	Increasing time reduced the formation of HbA1c and, thus, inhibited the production of glycated proteins	[13]
**3**	Saponin extract	Rabbit	Direct hypoglycemic agent	[25]
**4**	Fruit extracts	Rat	Glucose-stimulated insulin release	[72]
**5**	Fruit extracts	Rat	Lowered glycemia in short- and long-term experiments and during an oral glucose tolerance test (OGTT), lowered serum triglyceride concentration, prevention of a progressive increase in serum cholesterol concentration, increase in epididymal fat weight and lesser decrease in body weight	[74]
**6**	Seed extracts	Rat	Glucose homeostasis in rats injected with the β-cytotoxic agent and glucose-stimulated insulin secretion from rat-isolated pancreatic islets	[73]
**7**	Seed extracts	Rat	Decolorized DPPH, possessed antioxidant potential, decrease in serum glucose levels, and time-dependent decrease in blood glucose levels	[75]
**8**	Seed extracts	Rat	Direct action on endocrine pancreatic B cells	[76]
**9**	Fruit extracts	Rat	Positive effect in the treatment of diabetic neuropathy	[78]
**10**	Fruit extracts	Rat	Protective effect against cognitive impairments	[79]
**11**	Fruit extracts	Rat	Protective effect against liver/kidney	[81]
**12**	Fruit extracts	Rat	Protective effect against diabetic neuropathic pain	[82]
**13**	Seed extracts	Rat	Protective effect against pancreatic β-cell mass	[80]
**14**	Seed extracts	Rat	Reduction in aspartate aminotransferase (AST) and lactic dehydrogenase (LDH) and increase in blood levels of gamma-glutamyl transferase (GGT) and alkaline phosphatase (ALP)	[77]

## Data Availability

Not available.

## Sample Availability

Not available.
